# New products

**DOI:** 10.1155/S1463924699000127

**Published:** 1999


Journal of Automated Methods & Management in Chemistry, Vol. 21, No. 3 (May–June 1999) pp. 107–112

### New products

### Universal microplate handler

The product brochure for *Twister*, the universal microplate handler, is now available. *Twister* functionality, features, and productivity enhancements are described with general references to the microplate instrument market (without mentioning specific instruments). The list of available interfaces is given separately and frequently updated. Domestically, *Twister* Direct interfaces are sold through the Inside Sales Group and the developer's *Twister* is sold through the Drug Discovery Group. There are several OEM Twister interfaces available.

*Twister* software initiates the scientific instrument's method. After completion, *Twister* retrieves the plate from the instrument and places it into an output rack. This process is repeated, resulting in an unattended run. Twister's software is capable of cycling plates in predetermined intervals to accommodate kinetic applications. It features lid storage capability to minimize contamination. There is also both barcode reading and cycling software available as options.

*For more information contact Sharon Correia, Zymark Corporation, Zymark Center, Hopkinton, MA 01748, USA; Tel.: +1 508 435 9500; Fax: +1 508 435 3439; website http://www.zymark.com*

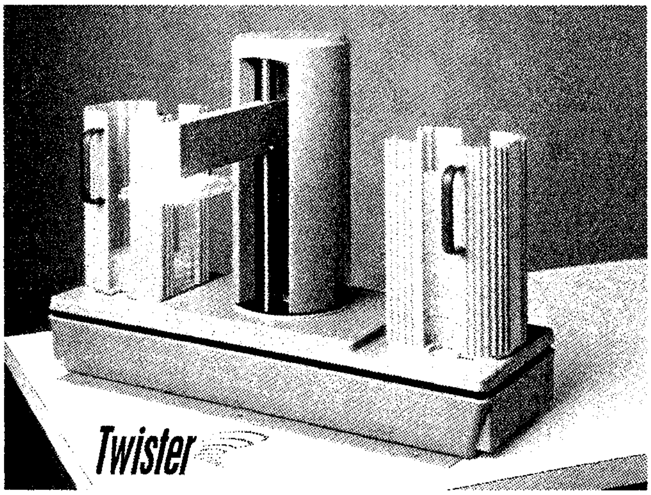

*A low cost, benchtop workstation designed to load and unload microplates on a wide variety of analytical instruments.*

### Shimadzu QP-5000/5050A GC/MS system

Thin Layer Chromatography (TLC) is a screening method used in many analytical areas. Complementary methods are then typically required in order to evaluate and confirm positive results. This is particularly so in the clinical laboratory—in drugs-of-abuse screening, for example. The additional sample preparation required can be time-consuming and labour-intensive for laboratories, which are already under pressure for higher throughput, greater efficiency and improved productivity.

The advantages of mass spectrometry are its simplicity, speed and the accuracy and reliability of the method. The Shimadzu QP-5000/5050A—a benchtop GC/MS system, with an extended mass range of 900DA—is equipped with a direct-sample inlet (DI-50) for the introduction of TLC sample fractions. A small amount of the selected sample spots on the TLC plate is scratched off and placed on the direct-inlet sample probe, introducing it to the mass spectrometer for heating, evaporation in the ionization chamber and measurement. The resulting thermogram provides an instant check of TLC screening results.

A GC column can be simultaneously installed to offer greater flexibility, without any downtime required for reconfiguration.

The use of the DI-50 with the Shimadzu QP-5000/5050A is not confined to clinical chemistry. It can be used in any application where TLC is used for screening and mass spectrometric confirmation of results is required—such as the analysis of solid matter in the polymer and pharmaceutical industries.

*For more information contact Shimadzu Europa (UK Branch), Mill Court, Featherstone Road, Wolverton Mill South, Milton Keynes MK12 5RE, UK; Tel: +44 (0)1908 552200; Fax: +44 (0)1908 552211; e-mail: Sales@Shimadzu.co.uk*

### New UV-Vis and FTIR software from Shimadzu

Two sophisticated new UV-Vis and FTIR software packages have been launched by Shimadzu, which contain all the functions required for validation, IQ/OQ and GLP in accordance with the European Pharmacopoeia.

HyperUV 1.50 and HyperIR 1.51 provide simple functions for the treatment and qualitative evaluation of spectra, as well as complex applications such as DIN H 18 analysis (oil in water) for FTIR. Functions for quantification, multi-component analysis and colorimetric measurements for UV-Vis are also included, as well as all normal functions for measurement and data processing. HyperUV 1.50 also provides a series of tests for routine validation of wavelength, potassium dichromate extinction, stray light measurement and resolution, which are in full accordance with the European Pharmacopoeia. After performing the tests, the software automatically generates a report which lists all test results and qualifies the overall result. HyperIR 1.51 enables manual or automatic validation for Shimadzu's new series FTIR-8000 (FTIR-8300 and FTIR-8700) systems. Again, test procedures accord with the requirements of the European Pharmacopoeia, which prescribes a polystyrene filter for tests. A built-in filter plate is used for automatic validation of wavelength accuracy and other prescribed tests. Both HyperUV and HyperIR software also provide data processing and GLP-specific functions, automatically documenting and recording measurement parameters, data and mathematical treatment required by GLP guidelines. Records can then be printed. In addition to user-specific and laboratory functions, the software also allows password-protected user groups to be set up. This enables the software options to be limited to only the required menus, commands and toolbars which the user will employ. It is also possible to modify the user interface and to generate toolbars with standard commands for routine analyses.

*For more information contact Shimadzu Europa (UK Branch), Mill Court, Featherstone Road, Wolverton Mill South, Milton Keynes MK12 5RE, UK; Tel: +44 (0)1908 552200; Fax: +44 (0)1908 552211; e-mail: Sales@Shimadzu.co.uk*

### Shimadzu VP HPLC

New validation and productivity—CLASS VP—software from Shimadzu enables HPLC users to achieve increased performance, reliability and accuracy from their analyser by simplifying and enhancing system diagnostics and validation.

The HPLC analyser incorporates advanced automation features including robotic autosampling from one- to 96-well or high density titer plates, an exclusive plug-and-play module system for optimum configuration and easy expandability, and easy fail-safe operation with built-in validation functions for confident compliance with cGMP and GaLP requirements. Now, as standard, analysers feature new 32-bit CLASS VP software which will check and validate all system functions and the entire system operation at the touch of a button, clearly displaying all results to assure the analyst of efficient validation at all stages. Times and effort required for instrument and operational certification is thereby reduced and downtime costs minimized. The VP run will also provide threshold values for the lifetimes of consumables, such as pump seals and pistons, detector lamps, and autosampler seals. Average standard values are listed, which are easily adjusted according to specific applications. The status of the entire instrument system is captured and clearly indicates when certain parts will need to be replaced.

The software includes a system suitability test—modular and total system—to ensure that all functions are performing at optimum efficiency. A simple, automatic, validation run will give the user all the information required to be sure of maximum performance—from series number up to a full list of any problems and malfunctions. Date and time, memory, pulse, flow, LPGE test program, leakage for autosampler and UV detector are included as part of the run. Flow and pressure compensation, leak sensor threshold, seal sensor threshold, seal life, operation mode, initialize parameter and change password functions are included under calibration.

*For more information contact Shimadzu Europa, Mill Court, Featherstone Road, Wolverton Mill South, Milton Keynes MK12 5RE, UK. Tel: +44 (0)1908 552200; Fax: +44 (0)1908 552211; e-mail: Sales@Shimadzu.co.uk*

### *Laboratory Equipment* magazine

*Laboratory Equipment*, published for over 35 years, reaches 135 000 buyers in the international laboratory industry. It serves industrial, government, university and independent laboratories. Readers include lab managers, scientist, chemists, and research and development specialists. *Laboratory Equipment* currently carries a 58% share of market business in the USA and has been the number 1 trade magazine in the USA for 10 years. Published by Cahners/Business Information/Reed Elsevier, it is the sister publication of *Laboratory Products International Brussels*.

The most popular issue of the year is the tenth corporate solutions issue. This enables companies to discuss products, missions, goals and capabilities.

*For more information and details on advertising contact Laboratory Equipment, First Floor, Premier House, 1 Cobden Court, Wimpole Close, Bromley, Kent BR2 9JF, UK. Tel: +44 (0)181 464 5577; Fax: +44 (0)181 464 5588; e-mail: stuart.smith@ssm.co.uk*

### Multi-channel cassette pump

Watson-Marlow Pumps have launched a new CA multi-channel cassette pump. The CA has many new features, including the following.

- Flow rate accuracy to 0.5%
- Fine 30 turn accurate occlusion scale
- Separate cassette release lever
- Lockable occlusion setting
- New sprung track cassette
- Flow rates from 0.6 $\mu l\ min^{-1}$ to 42 $ml\ min^{-1}$
- Full flexibility from four to 48 channels
- Independent flow rate for each channel

With a choice of drives to offer high accuracy control and dispensing, the CA is ideal for many multi-channel applications. The new pump cassettes feature a sprung track which ensures the ideal setting in the majority of applications. If required, the setting can be optimized using 30 turn scale to control the flow and pressure characteristics. The occlusion adjustment is lockable to prevent tampering and maintain settings during the tube changes. The sprung track also helps to reduce pulsation by 25% compared with other cassette pumps and maximizes tube life.

Designed for simplicity and ease of use, the cassettes locate in a track to ensure correct alignment at all times—even when the pumphead is not full of cassettes. The CA cassette also gently pre-stretches the tubing to

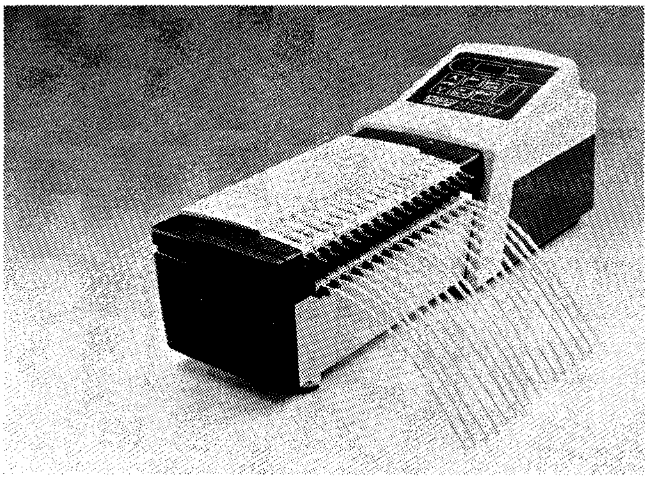

*The Watson-Marlow 205/CA. The cassette pump is used to help determine the large scale effects of industrial waste on the environment, at the Zeneca Brixham Environmental Laboratory.*

hold it in place during loading, and, together with a dedicated release lever, it ensures trouble-free tube loading.

The CA pumps accept tubing from 0.13 to 2.79 mm bore, offering flow rates from 0.6 μl $min^{-1}$ to 42 ml $min^{-1}$. Liquid is contained completely with the tube, so there is no contamination of either the pump or fluid. Pumpheads can be fitted to a range of drives to provide optimum flexibility with up to 32 channels on Watson-Marlow's 205 drives and 48 channels on 500 series drives. With the option to add channels, this flexibility allows for growth as applications dictate.

Watson-Marlow's 205 drives offer manual or analogue auto control, whilst the 500 series drives offer manual control, auto control by either analogue or digital RS232 signals and dispensing options.

*For more information contact Mark Rawet, Marketing Manager, Watson-Marlow Ltd, Falmouth, Cornwall TR11 4RU, UK. Tel: +44 (0)1326 370 370; Fax: +44 (0)1326 376 009; e-mail: markr@watson-marlow.co.uk; http://www.watson-marlow.com*

### Prima upgrade offers greater flowrate choice and quieter operation

USF Elga, pure water specialists, have increased the flowrates for its Prima 1, 2 and 3 models, which produce primary grade water for laboratory, medical and industrial applications.

The models now offer maximum flowrates of 7–21 l/h at 15 °C, compared with the previous 4–16 l/h at 25 °C.

Customers requiring larger quantities of general laboratory grade water can opt for the Prima 4, 5, 6 and 7, which continue to offer flowrates of 30–120 l/h.

All Prima models now incorporate a space-saving, quieter integral boost pump, providing a more pleasant working environment for laboratory staff.

They use proven reverse osmosis technology to produce primary grade water which is ideal for feeding Elga's Maxima or UHQ-PS units. The water quality is also suitable for general laboratory applications, e.g. glassware washing, and feeding autoclaves and environmental cabinets.

Elga products are all backed up by a strong commitment to customer care, including technical support from a nationwide network of specialist engineers.

*For further information please contact Andrew Webber. Tel: +44 (0)1494 887 835; Fax: +44 (0)1494 887 824*

### AEA Technology engineering software adds 'accumulated knowledge' data management solution

*AXSYS provides enterprise-wide engineering database for use across the process industries*

**Calgary, Canada, 13 October 1998:** AEA Technology Engineering Software announced today that it is expanding its integrated process engineering systems to include an engineering knowledge database for deployment throughout the lifecycle of complex chemical process plants. AXSYS, which was initially developed as PlantCONCEPT by EA Systems (Alameda, CA), will be marketed and sold by EA Systems and Hyprotech, both AEA Technology companies.

AXSYS optimizes enterprise workflow and enables collaborative engineering and early validation of plant design by linking process simulation and project evaluation data in a common database repository. This has been shown to reduce the time to create a process design package by more than 50%, resulting in superior plant and process design. AXSYS is the only integrated software application that automatically generates both PDFs (Process Flow Diagrams) and P&IDs (Piping and Instrumentation Diagrams) based on a rule-based system and best practices.

'AXSYS is the first software to integrate process simulation and accumulated engineering knowledge for access across the enterprise', said Steve Curl, Managing Director of AEA Technology Engineering Software. 'It is the key to a fully integrated engineering work process that draws on AEA Technology's comprehensive portfolio of solutions for the process plant design and operations lifecycle, providing owner-based operators with an opportunity to improve their competitive position'.

'The use of AXSYS enables process engineers to rapidly prototype and evaluate multiple design alternatives early on in the plant creation process to determine the most cost-effective and efficient solutions', said Ken Adamson, Vice President of EA Systems. 'It eliminates redundant data input, reduces errors caused by inconsistent data entry and dramatically shortens the design cycle'.

AXSYS provides a comprehensive front-end process design environment, with integrated links to HYSYS and other process simulation software, cost estimating applications, e.g. ICARUS 2000, and heat exchanger design calculations, e.g. HTFS. It automatically develops PFDs based on configurable design rules and logic, creates heat and material balance tables, and automatically generates initial P&IDs. It also provides seamless integration with Microsoft Excel and other standard desktop applications for data entry and reporting. AXSYS data can be easily shared over the web.

Through its link to the EA Systems' PASCE® software suite for 2D intelligent schematics and 3D plant modelling, AXSYS enables process engineers to incorporate 3D layout data for detailed equipment design, 3D modelling, and visualization and drawing generation. The integration capability with HYSYS capitalizes on the simulation environment through iteration of design information for greater accuracy and detail. These results are already being achieved by major chemical companies through the use of AXSYS technology.

AEA Technology Engineering Software includes Hyprotech, EA Systems, CFX, HTFS, SPS and nCode, and is a division of AEA Technology plc, a major international science and engineering company. AEA Technology supplies innovative technical, safety and environmental solutions to a broad range of industries worldwide. AEA Technology employs nearly 4500 people in 26 locations around the world. AEA Technology brings science to the marketplace through consultancy, technical services, hardware and software systems, research and development and technology transfer.

*For more information, visit AEA Technology's web site at http://www.aeat.com or contact Liz Filmer, EA Systems (Europe) at: Lenton Business Centre, Lenton Boulevard, Nottingham NG7 2BY, UK. Tel: +44 (0)115 978 3127. http://www.easystems.com, Marie Telepneff, EA Systems at +1 510-748-4855, Tim Fox, Hyprotech at +1 403-520-6000 http://www.hyprotech.com*

### PAAR Scientific newsletter details new products and applications

PAAR Scientific has published a newsletter detailing new products and applications in the process, food and drink, and general manufacturing industries.

Featured is a new bench-top density meter for liquids and gases offering many advanced features to meet the demands of the 21st century. Their new pressure measuring cell, UDS 200, is designed to determine all rheological data, and an application story of finding the 'jellification' point is good reading. Digital spirit gauging at the Grand Chartreuse Carthusian Monastery is carried out with PAAR's DMA 5000 density meter. Ascertaining the $CO_2$ concentration of carbonized drinks is reduced to a simple 10 s measuring cycle by Carbo2000, an automatic analyser developed by PAAR.

Coca-Cola keeps costs down using PAAR's COBRIX2 diet or Brix concentration and $CO_2$ content of each delivery. Randals Batteries invested in a DMA 35 hand-held density and concentration meter, and found it to be the most used tool in their workshops.

*For further information contact Bryan Treherne at PAAR Scientific Ltd*

### New product/Agency for Metrohm UK

*'Versatile TOC does TN as well'*

The new Thermalox range of windows-driven analysers can analyse a combination of the following depending on the analyst's requirements: TOC, TIC, TC, NPOC, TN, NN, TON.

A common requirement is for TOC and TN performed simultaneously on the same sample. This is achieved using catalytic thermal oxidation to convert the carbon and nitrogen to $CO_2$ and $NO_x$, respectively, which in turn are detected by the ultra-sensitive NDIR detector.

For those analysts who are interested in nitrogen alone, a single analyser can analyse for total nitrogen (TN), nitrite and nitrates (NN), and total organic nitrogen (TON), which is equivalent to Kjeldahl nitrogen.

The NN is analysed by passing the the sample through a reactor containing a reducing reagent, the resulting NO is picked up by the NDIR detector.

The TON is calculated by subtracting the NN from the TN.

The Thermalox range complies fully with the new British and European standard for TOC (BS EN 1484), especially with regard to proper calibration and complete oxidation of all organic material.

*For further information please contact Metrohm UK Ltd. Tel.: +44 (0)1280 824 824; Fax.: +44 (0)1280 824 800; e-mail: enquiry@ metrohm.co.uk*

### MDC Technology establish US operation, Aspen Technology and Honeywell face new competition

*Acquisition of Profitpoint Solution Inc establishes US sales and project capability*

**Teesside, UK—(Business Wire) 8 October 1998:** MDC Technology, a leading provider of Advanced Process Control and Optimization technology to the process industries, today announced that it has executed an agreement to acquire Profitpoint Solutions, Inc. Based in Houston, Profitpoint Solutions offers consulting and implementation services for advanced manufacturing automation, execution and information technology applications in the process industries.

This strategic acquisition reinforces MDC Technology's commitment to the North and South American market, and establishes advanced process control (APC), and real-time optimization (RTO) and information system (IS) execution capability in this region.

This acquisition marks another significant step in the development of MDC Technology's capabilities for delivering the best in class APC/RTO and IS solutions to the North and South American market. In doing so, MDC Technology will provide customers with an alternative to Aspen Technology and Honeywell Hi-Spec Solutions.

'We have proved that on a global basis, MDC has competed successfully in delivering its solutions. We believe our commitments to customer satisfaction, reputation of being easy to do business with, state-of-the-art product portfolio and ability to deliver fast track commercially attractive solutions have all been key factors to our success', commented Alan Dormer, Managing Director of MDC Technology. 'It is exactly these principles upon which MDC Technology Inc. will deliver solutions to the North and South American market'.

Dr Douglas C. White has been announced as President of MDC Technology Inc. Dr White was the president and founder of Profitpoint Solutions Inc. Previously he was Senior Vice President for Automation Technology for Aspen Technology Inc. He joined Setpoint Inc. in 1979 and was its President from 1993 through the time of its purchase by Aspen Technology Inc. in 1996. Setpoint was well known for its leading technology developments and implementation services in advanced manufacturing automation and information technology.

Dr White is a well-known expert in advanced automation and information technology applications with more than 20 years of international experience designing, developing and installing state-of-the-art systems. He has authored or co-authored more than 30 technical articles in the field, many documenting first of a kind installations.

Commenting on the acquisition, Dr White said, 'We believe there is today a real need in this market for a company that is focused on customer satisfaction with technology that is easy to install and maintain and continues to provide business value year after year. I have been impressed with MDC Technology's dedication to these ideas and the business success they have achieved. We are sure that clients in North and South America will soon come to know and appreciate this new, and we believe refreshing, approach to business'.

*About MDC Technology*

MDC Technology are a leading worldwide supplier of real-time optimization (RTO), advanced process control (APC) and information systems (IS) to the oil & gas, oil refining, gas processing, petrochemicals, chemicals and power generation industries. Our solutions provide benefits of c. 5–10% increase in profitability to the operation of our customers' processes.

MDC Technology's best in class product portfolio comprises the following.

- Real-time optimization. RTO+ and HYSYS.RTO+ are the industry's leading real-time optimization systems.
- Advanced process control. SMOC III is the most technically advanced multi-variable optimizing controller available and has the second largest installed base in excess of 400 applications.
- Information systems. PI from OSI Software Inc. is the industry's leading real-time information system.

*For further information about MDC Technology, visit out website at http://www.mdctech.com*

**TurboSEC™ Size exclusion chromatography software**

New from Perkin-Elmer, TurboSEC™ is an optional 32-bit software package that provides complete and flexible molecular weight distribution processing and reporting of TurboChrom Client–Server and TurboChrom Workstation data. Methods may be run automatically, as part of a TurboChrom sequence to generate size exclusion software reports and plots at the completion of each data acquisition run.

*For further information, contact Perkin-Elmer Limited, Post Office Lane, Beaconsfield, Bucks HP9 1QA. Tel.: +44 (0)1494 676161; Fax.: +44 (0)1494 679331/3; e-mail: greenca@eur.perkin-elmer.com; Internet http://www.perkin-elmer.com*

**Environmental applications of GC/MS**

Three new application notes, available from Perkin-Elmer, discuss the use of GC/MS in water analysis. Methodology and results are outlined and illustrated for the following applications.

- Analysis of organo-phosphorus and organo-nitrogen herbicides in water, using GC/MS. Precision using selected ion recording (SIR) in the ppb concentration range.
- Determination of VOCs in water, using static head-space GC/MS. Sensitivity and precision using simultaneous full scan and selective ion recording.
- Determination of organochloride pesticides in water using GC/MS. Enhanced sensitivity in full scan mode using large volume injection (LVI).

*For further information, contact Perkin-Elmer Limited, Post Office Lane, Beaconsfield, Bucks HP9 1QA. Tel.: +44 (0)1494 676161; Fax.: +44 (0)1494 679331/3; Internet http://www.perkin-elmer.com*

**Spectrum One FT-IR systems—flexible, user-friendly technology**

Perkin-Elmer's new Spectrum One™ is the latest addition to the Spectrum FT-IR family of FT-IR spectrometers, designed to give the highest quality results in the quickest and most straightforward manner. This instrument will be particularly useful for routine testing and trouble shooting in quality assurance laboratories.

This simplicity of use is achieved using IR Assistant™ question/answer wizard software, automatic accessory recognition, and the Look-Ahead facility (which eliminates the need for the user to collect background scans). Data quality can be assured by using the IR Expert option, which alerts the user to possible spectral errors. Automatic atmospheric absorption suppression also optimizes data quality, by removing the effects of water vapour and carbon dioxide from sample spectra.

The Spectrum One can be controlled either by PC, or by the unique built-in touch screen, which may be used to run an entire analysis, from sample to report, following a pre-programmed method.

*For further information, contact Perkin-Elmer Limited, Post Office Lane, Beaconsfield, Bucks HP9 1QA. Tel.: +44 (0)1494 676161; Fax.: +44 (0)1494 679331/3; e-mail: greenca@euro.perkin-elmer.com; Internet http://www.perkin-elmer.com*

### FT-NIR spectroscopy for the wine industry

Tartrates, or 'finings', are used in the wine industry to clear muddiness or sediment prior to bottling. Among the different types of finings used, many are the by-products of other processes and are often of extremely variable quality, so that verification is important. A new application note available from Perkin-Elmer describes the use of FT-NIR spectroscopy to discriminate between two typical samples, based on calcium tartrate and potassium bitartrate. NIR reflectance is an ideal method for finings analysis as no sample preparation is involved, and both chemical and physical property information can be determined. Instrumentation and methods are discussed, and sample spectra are illustrated.

*For further information, contact Perkin-Elmer Limited, Post Office Lane, Beaconsfield, Bucks HP9 1QA. Tel.: +44 (0)1494 676161; Fax.: +44 (0)1494 679331/3; e-mail: greenca@eur.perkin-elmer.com; Internet http://www.perkin-elmer.com*

### FT-NIR discrimination of celluloses for the pharmaceutical industry

New application notes from Perkin-Elmer describe the use of the IdentiCheck FT-NIR spectrometer to discriminate between different cellulose raw materials used in the pharmaceutical industry.

*The discrimination of celluloses by FT-NIR spectroscopy using soft independent modelling by class analogy (SIMCA)*

Celluloses were sampled using a diffuse reflectance fibre optic probe, and were found to be spectroscopically very similar. They could be readily discriminated using SIMCA. Details of the model are discussed, with the methods used, and illustrations of results.

*A comparison of fibre optic and ICRA sampling for SIMCA classification of different celluloses*

As before, the same celluloses were sampled using a diffuse reflectance fibre optic probe and were readily discriminated using a SIMCA model. The procedure was repeated using the fixed, fibreless IndentiCheck Reflectance Accessory (ICRA), which provided superior data to that collected using fibre optics. Details and illustrations are given of the methods used and results obtained.

*For further information, contact Perkin-Elmer Limited, Post Office Lane, Beaconsfield, Bucks HP9 1QA. Tel.: +44 (0)1494 676161; Fax.: +44 (0)1494 679331/3; e-mail: greenca@eur.perkin-elmer.com; Internet: http://www.perkin-elmer.-com*

### FT-NIR spectroscopy for moisture and protein determination

A new application note available from Perkin-Elmer describes the use of FT-NIR spectroscopy to determine, with an estimated prediction error of less than 0.5%, the protein and moisture content in ground wheat raw materials, as used in the agricultural industry. Instrumentation and method are outlined, and results are illustrated, for the analysis of 70 samples of ground wheat.

NIR spectroscopy is widely used at various stages of a range of manufacturing processes, particularly for raw material qualification and quantitation. The technique offers a fast and reliable alternative to traditional quantitative methods, which often take many hours to provide results.

*For further information, contact Perkin-Elmer Limited, Post Office Lane, Beaconsfield, Bucks HP9 1QS. Tel.: +44 (0)1494 676161; Fax.: +44 (0)1494 679331/3; Internet http://www.perkin-elmer.com*

